# Epstein-Barr virus positive peripheral T cell lymphoma with novel variants in *STAT5B* of a pediatric patient: a case report

**DOI:** 10.1186/s12885-018-4311-z

**Published:** 2018-04-03

**Authors:** Zihang Chen, Limin Gao, Mi Wang, Yuan Tang, Sha Zhao, Weiping Liu

**Affiliations:** 1 Department of Pathology, West China Hospital, Sichuan University, No. 37 Guo Xue Xiang, Chengdu, 610041 Sichuan China; 2 Department of Dermatology, West China Hospital, Sichuan University, No. 37 Guo Xue Xiang, Chengdu, 610041 Sichuan China

**Keywords:** EBV, PTCL, Pediatric, *STAT5B*

## Abstract

**Background:**

Epstein-Barr virus positive peripheral T cell lymphoma (EBV + PTCL) is a rare type of lymphoproliferative disorder which is always present in late adulthood. However, pediatric EBV + PTCL is extremely rare and always present with lymphadenopathy. Additionally, gene detection was not performed in all of these pediatric patients.

**Case presentation:**

We report an EBV + PTCL in a 9-year-old child with initial symptom of subcutaneous masses without lymph node involvement. Histologically, the neoplastic cells were centroblastoid with round or oval nuclei, slightly condensed chromatin and median eosinophilic inconspicuous nucleoli. Immunohistochemically, all neoplastic cells were positive for CD8, GranzymeB and TIA-1. Two novel variants (S420Y and E623K) were detected in *STAT5B*.

**Conclusion:**

To the best of our knowledge, this is the first case of EBV + PTCL with STAT5B variants of a pediatric patient presented as extranodal lesions.

**Electronic supplementary material:**

The online version of this article (10.1186/s12885-018-4311-z) contains supplementary material, which is available to authorized users.

## Background

Peripheral T cell lymphoma, not otherwise specific (PTCL-NOS) is the most common type of T cell lymphoma in adulthood which is account for 25.9% in Western countries [1]. However, as a variant of PTCL-NOS, Epstein-Barr virus-positive peripheral T cell lymphoma (EBV + PTCL) is a rare type which mainly occurs in the elderly with common primary site of lymph nodes and aggressive clinical course [2]. Additionally, for pediatric patients, anaplastic large cell lymphoma (ALCL) is the most common type non-Hodgkin lymphoma while PTCL-NOS only occupied 1.3% of the patients with non-Hodgkin lymphoma. Although EBV- associated T/NK lymphoproliferative disorder also occupied a large part in Asian pediatric patients [3], EBV + PTCL is extremely rare, with only 4 patients (including this case) of childhood have been reported [4, 5]. Among these patients, the primary sites were lymph nodes with or without extranodal involvement, and the genetic detection was not performed. We herein report a case of this rare lymphoma with novel *STAT5B* variants, multiple subcutaneous masses as initial symptom without lymph node involvement in a 9-year-old patient.

## Case presentation

### Medical history

A 9-year-old boy was admitted to pediatric department with chief complaining of multiple subcutaneous masses for 2 months. During this period, the patient had no fever, lymphadenopathy or hepatosplenomegaly. The patient had a history of Nuss procedure for funnel chest 7 years ago and diagnosed with microsomia 3 years ago. The patient was treated with growth hormone for 6 months after diagnosis with an outcome of 2 cm growth of stature. His family history was unremarkable for early-onset cancers or other hereditary diseases.

**Fig. 1:**
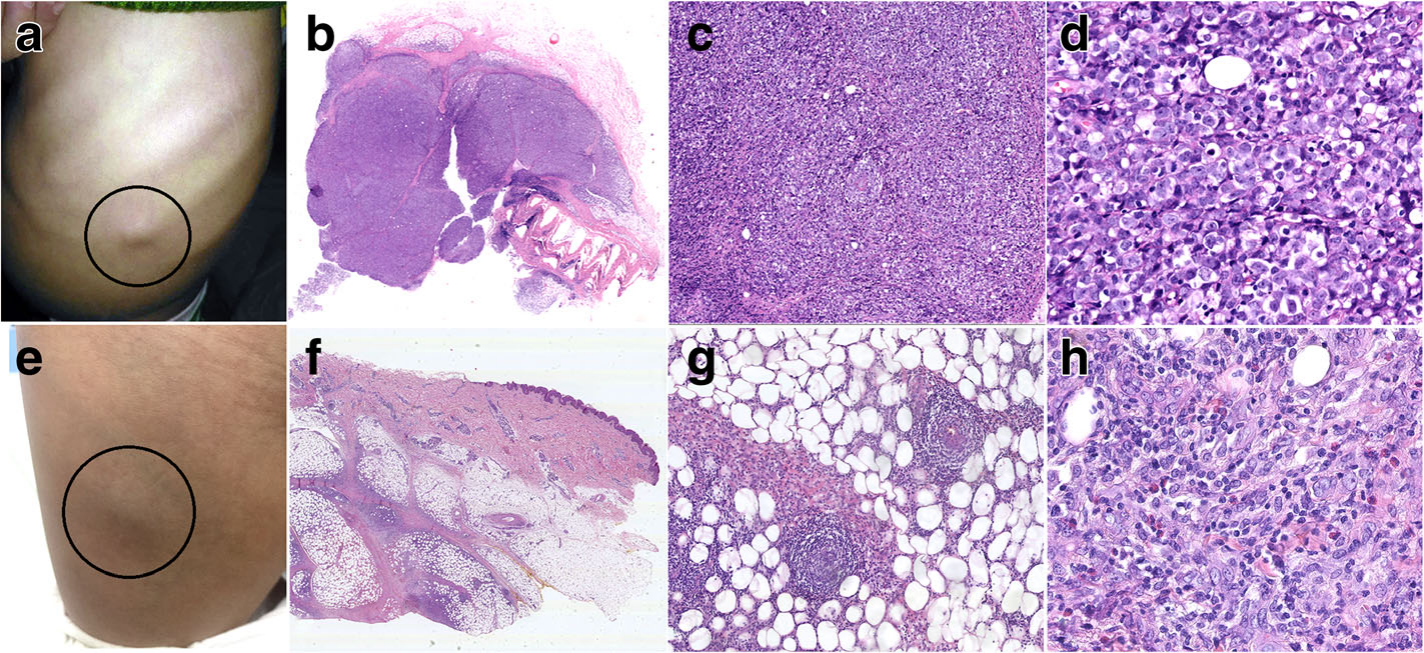
Subcutaneous masses and microscopic appearance of the tumor in de novo and relapse. **a** Subcutaneous mass in left hypochondrium (de novo); (**b**): The neoplasm is restricted in the subcutaneous tissue, HE× 1 (de novo); (**c**): Vascular infiltration, HE× 200 (de novo); (**d**): Medium to large-sized atypical lymphocytes in mosaic arranging, HE× 400 (de novo); (**e**): Subcutaneous mass in right thigh (relapse); (**f**): The neoplastic cells extend into subcutaneous tissue as panniculitis-like change without corium involvement, HE× 1 (relapse); (**g**): Vascular infiltration, HE× 200 (relapse); (**h**): Small to medium-sized atypical lymphocytes with a few eosinophils infiltration, HE× 400 (relapse)

The physical examination showed body height of 110 cm (<-3 standard deviations, SDS) with multiple subcutaneous masses located in shoulders, nape, submandibular regions and left hypochondrium (Fig. 1a), but the spleen and liver were impalpable. The blood routine test revealed the white blood cell count of 14.3 × 10^9/L (reference range 3.5–9.5 × 10^9/L), platelet count of 417 × 10^9/L (reference range100–300 × 10^9/L) and hemoglobin of 101 g/L (reference range115-150 g/L). The lactate dehydrogenase (LDH) level was measured as 264 U/L (reference range 35-105 U/L) while the EBV-DNA was detected of 418 copies/ml in serum. The insulin-like growth factor-1 (IGF-1) load in serum was only 79.6 ng/ml which was far lower than the low-normal level. An excisional biopsy of subcutaneous mass in the abdomen had been performed after admission.

### Pathologic findings

**Fig. 2:**
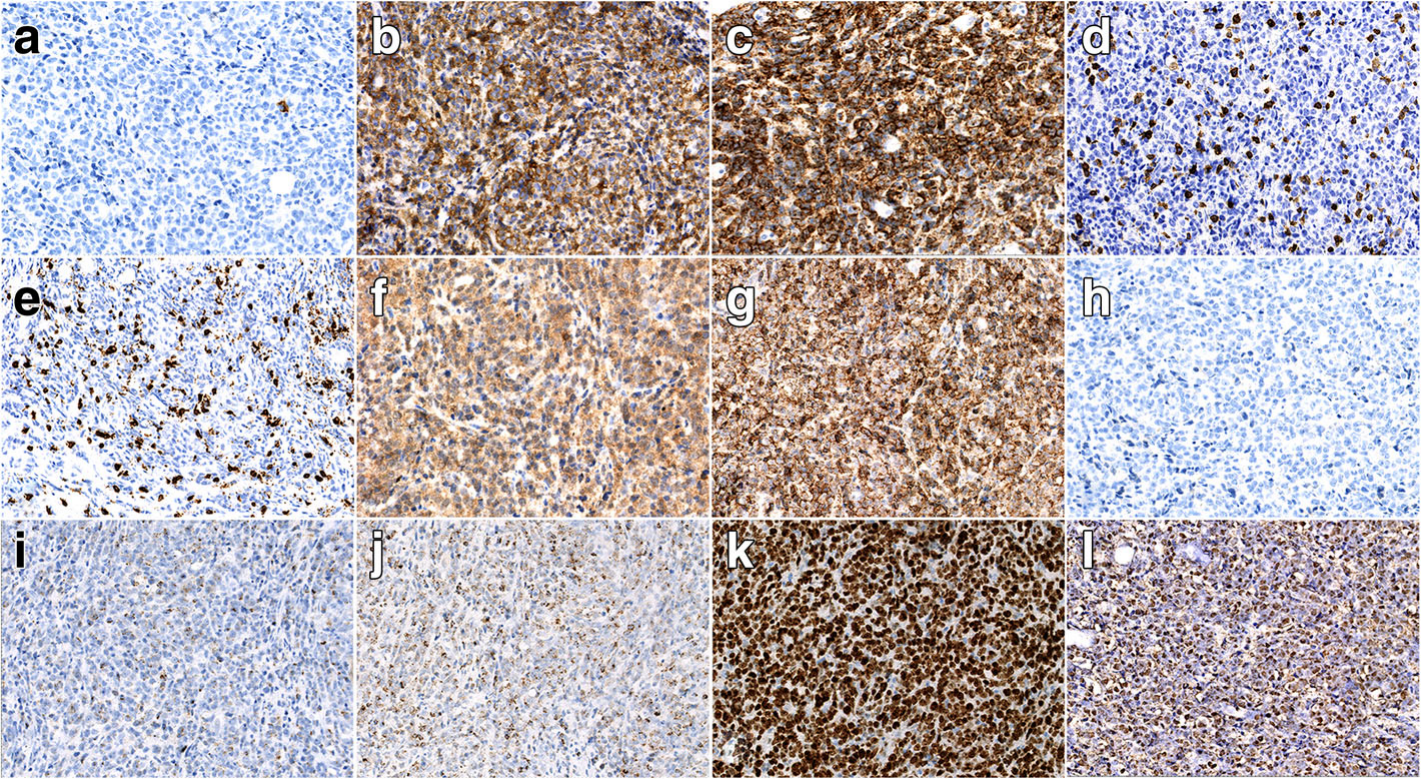
Immunophenotypes and EBER-ISH of de novo lymphoma. **a** CD20; (**b**): CD3p; (**c**): CD2; (**d**): CD5; (**e**): CD7; (**f**): CD4; (**g**): CD8; (**h**): CD56; (**i**): Granzyme B; (**j**): TIA-1; (**k**): Ki-67/MIB-1; (**l**): EBER-ISH

Microscopically, it appeared that medium to large-sized atypical lymphocytes proliferated and diffusely infiltrated into subcutaneous tissue in mosaic arranging, with round or oval single nucleus, punctate nuclear chromatin and visible nucleoli, numerous mitotic figures; partly with lobular nuclei (Fig. 1b, c and d), slightly condensed chromatin and central eosinophilic inconspicuous nucleoli, high nucleocytoplasmic ratio, numerous mitotic figures and vessel invasion. Many small lymphocytes were appeared in the background with scattered tumor giant cells. Immunohistochemically, the neoplastic cells were CD2+, CD3p+, CD8+, CD30+, Granzyme B+, TIA-1+, CD5-, CD7-, CD4-, CD56-, MPO-, TdT-. The proliferation index was approximately 80%, evaluated by Ki-67 staining. and the result of EBER-ISH appeared positive for most of the atypical cells (Fig. 2). Monoclonal TCR-γ gene rearrangement was detected by the Polymerase Chain Reaction heteroduplex analysis (PCR-HA) and gene-scanning (Additional file 1: Figure S1). In conclusion, the final diagnosis was made as EBV + PTCL.

### *STAT5B* variant investigation

**Fig. 3:**
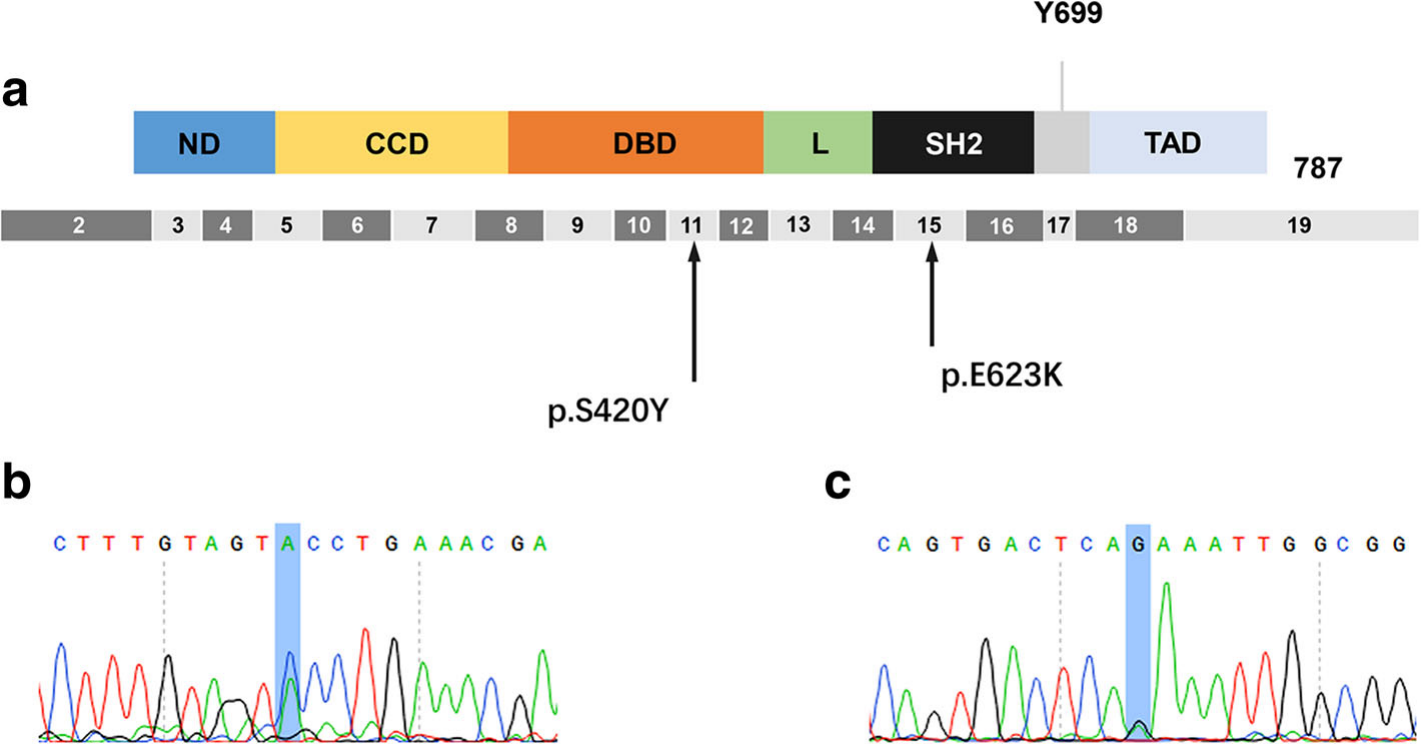
Structure and variants of *STAT5B*. **a** STAT5B variants identified in neoplastic tissue of this patient, in exon 11 and exon 15. **b** Exon 11: c.1259C > A, p.S420Y; (**c**): Exon 15: c.1867G > A, p.E623K

STAT5B variant analysis was performed on both formalin-fixed paraffin-embedded (FFPE) neoplastic tissue and blood sample by sanger sequencing (Additional file 2). In the neoplastic tissue, 2 variants (S420Y and E623K) were detected in *STAT5B* (Fig. 3), however, such variants were not found in blood sample.

### Treatment and follow up

After diagnosis, a positron emission tomography–computed tomography (PET-CT) was applied and revealed an increasing 2-Deoxy-2-fluoro-D-glucose (18F-FDG) uptake of multiple masses in subcutaneous tissue and muscles but no lymph node was involved (Additional file 3: Figure S2A). The patient showed impalpable subcutaneous masses after 2 cycles of chemotherapy (SMILE: Methotrexate, Ifosfamide, Dexamethasone, Etoposide, L-asparaginase), however, a second PET-CT was ordered 26 days later after chemotherapy which implied the relapse since the Deauville 5-point score (DS) was 5(Additional file 3: Figure S2B). Therefore, the protocol had been switched to more aggressive as modified SMILE regimen, leading to a complete remission after 8 cycles of chemotherapy (Additional file 3: Figure S2C). However, 3 months after the treatment, another subcutaneous mass was palpable in the right thigh and the excisional biopsy suggested relapse (Fig. 1e, f, g and h).

## Discussion

PTCL-NOS is the most common type of mature T-cell lymphoma in Western countries, and it is also the second most common mature T-cell lymphoma in China which is accounting for 13.2% of the patients [6]. In addition, this kind of lymphoma is always in adults but very rare in children. Scattered cohorts with a few cases have been reported in pediatric patients. A SNOMED search of the West China Hospital surgical pathology database from January 2014 to October 2017 identified 271 PTCL-NOS patients, whereas only 2 cases (0.7%, including this case) were detected in pediatric group (under the age of 14) during this period. Additionally, 14 of the 271 patients (5.1%) were measured as EBV + PTCL while only this case did not present with lymphadenopathy.

EBV + PTCL, as a variant of PTCL-NOS, is a rare malignancy which is more frequent in the late adulthood with high clinical stage and aggressive clinical course [2]. however, for the patients of childhood, it was extremely rare with limited cases have been reported (Additional file 4: Table S1) [4, 5, 7]. For these cases, lymph node involvement was the significant feature with the common site of cervical region. However, a few extranodal organs may also be involved, with lymphoma presenting in bone marrow and spleen [6]. Typically, the morphology of PTCL-NOS appears a mixture of small to large atypical lymphocytes, with clear cytoplasm, irregular nucleus and obvious nucleoli, invading micrangium and normal lymph node structure, but EBV + PTCL shows different cytomorphology—more commonly centroblastoid, often anaplastic, or plasmacytoid [8]. For the immunophenotype, PTCL usually loss the pan T-cell antigens, such as CD2, CD5, or CD7, of which, CD2 is the most stable T-cell marker. Moreover, most cases of EBV + PTCL are CD8+/CD4-, while double negative or CD4+/CD8- cases are less common, each accounting for about 15% of the cases [2]. Besides, cytotoxic molecule including GranzymeB and TIA-1 are expressed on nearly all of the cases (97%) [2]. In our study, the cytomorphology and phenotypes of the neoplasm conformed to EBV + PTCL with CD2+, CD3p+, CD8+, Granzyme B+, TIA-1+, CD4-, CD5-, CD7- centroblastoid neoplastic cells, nevertheless, the young age at onset, multiple subcutaneous masses without lymph node involvement and good general condition of the patient are different.

The immunophenotype of our young patient with CD3ε+, TIA-1+, GB+ and the positive EBER-ISH could raise the consideration of systemic EBV-positive T cell lymphoma of childhood. Moreover, a Korean study reported that chronic active EBV infection and systemic EBV-positive T cell lymphoma of childhood were the most common type of EBV-associated lymphoproliferative disorder among Asian teenagers [3]. Morphologically, these diseases appear to be polymorphic or monomorphic without obvious pleomorphism of infiltrated cells [9]. Besides, these patients always have a history of initial or chronic EBV infection and present with fever, lymphadenopathy, pancytopenia, progressive hepatosplenomegaly, multiorgan failure, more frequently developing to hemophagocytic lymphohistocytosis (HLH) [1]. However, the morphology and clinical features of our patient did not conform to such disease entitled systemic EBV+ T-cell lymphoma of childhood.

Extranodal NK/T cell lymphoma(ENKTL) should be taken into consideration as one of the differential diagnosis because they share many similarities in extranodal involvements, EBV association and immunophenotypes. Moreover, ENKTL occupies the largest proportion of mature T/NK cell lymphomas in western China [6]. ENKTL with cutaneous involvement or primary cutaneous ENKTL demonstrate atypical small to medium-sized lymphocytes with NK cell phenotype infiltrating into corium and extending into the subcutis, commonly with zonal necrosis. Therefore, ulcer and violaceous plaques are always presented on the skin [10]. Unlike this condition, in our case, the tumour was restricted to subcutaneous tissue without corium infiltration and necrosis. Besides, the tumour was originated from T cell linage, which was also different from ENKTL. Although previous studies reported that some ENKTL cases express TCR and were regarded as T-cell origin, it was rare and only accounted 11% of the cases in a recent study [11], however, most of the ENKTL of T-cell type are originated from γδT cells which rarely express CD8. The differences in systemic EBV + T-cell lymphoma of childhood, ENKTL and our case were summarized in Additional file 5: Table S2).

*STAT5B* gene consists of 19 exons with 77.23 kilobases (kb) span, located on chromosome 17q11.2. It encodes a slightly smaller peptide of 787 amino acid residues with 6 protein domains, N-terminal domain(ND), Coiled-coiled domain(CCD), DNA binding domain(DBD), Linker(L), Src-homology 2(SH2), Transactivation domain(TAD). *STAT5B* has been demonstrated to have strong correlation to growth by regulating growth hormone -insulin-like growth factor − 1 (GH-IGF-1) axis via activating IGF-1 expression [12]. A previous study reviewed 10 patients of growth failure due to *STAT5B* mutations indicated that severe growth failure, marked IGF-1 deficiency and insensitivity to GH are the essential clinical features [13]. This patient was at the height of 110 cm(<-3SDS) [14] with low IGF-1 in serum(<-2SDS) [15] and growth hormone insensitivity (growing 2 cm after 6-month growth hormone therapy, much lower than normal therapeutic response) that might arise our consideration of the abnormality of STAT5B signaling pathway. Moreover, the mutation of STAT5B has been detected in many malignancies, especially in γδ-T-cell lymphoma [16]. In a recent study, STAT5B has been measured in 51 ENKTL cases, to find 1 case of N642H and 2 cases of Y665F mutations [16]. However, no studies focus on STAT5B mutation in systemic EBV + T-cell lymphoma of childhood and PTCL-NOS was published. The mutation, N642H in Src-homology 2 (SH2) domain, was demonstrated to associate with increased phosphorylated protein and high proliferative activity of the tumor cells [16]. Accordingly, *STAT5B* sequencing was applied for this patient in both neoplastic tissue and peripheral blood sample. Interestingly, two variants which have not been reported, S420Y in DNA binding domain and E623K in SH2 domain, were detected in neoplastic tissue, however, such variants were not detected in blood sample. This result indicated that growth retardation of this patient was not caused by *STAT5B* signaling pathway dysfunction, but the variants of STAT5B (especially E623K in SH2 domain) in neoplastic tissue might contribute to the tumor proliferation. To the best of our knowledge, no researches investigated the STAT5B variant in PTCL-NOS. Our study may serve as a modest spur to induce someone to come forward with his valuable contributions.

Comparing to other EBV + PTCL, our patient showed different characteristics. EBV + PTCL is always associated with poor prognosis with the median survival of 4 months due to the drug résistance. However, our patient responded well and achieved complete remission after chemotherapy. Interestingly, this patient suffered relapse twice within 8 months.

## Conclusion

In conclusion, we reported a rare EBV + PTCL present in a growth retardation child with many unique features: 1. A young onset age at 9 years old 2. Primary subcutaneous infiltration without lymph node involvement. 3. Disseminated biological behavior of the lymphoma with unmatched clinical condition. 4. Novel detected variants (S420Y and E623K) in *STAT5B*. This case also has educational significance for both pathologists and hematologists. It may arise the awareness of applying for EBV detection for pediatric patients without lymphadenopathy diagnosed as PTCL-NOS in good general condition.

## Additional files

Additional file 1: **Figure S1.** IgH, IgK and TCR rearrangement. This figure shows results of IgH, IgK and TCR rearrangement. A: IgH and Igκ testing: No obvious clonal peak was detected (The peak in blue is normal according to the testing instruction). B: TCRγ testing: Clonal peak was detected (The peak in green). (TIFF 4875 kb)

Additional file 2: *STAT5B* variant investigation by Sanger Sequencing. This file provides the method of *STAT5B* variant investigation by Sanger Sequencing in details. (DOCX 13 kb)

Additional file 3: **Figure S2.** PET-CT images of the patients. This figure shows PET-CT results of the patient (pre-treatment, after 2 cycles of chemotherapy and after 8 cycles of chemotherapy). A: Pre-treatment PET-CT showed multiple lesions in subcutaneous tissue, muscle et al. B: PET-CT after 2 cycles of SMILE regimen, DS = 5. C: PET-CT after 8 cycles of modified SMILE regimen, complete remission. (TIFF 5450 kb)

Additional file 4: **Table S1.** Summary of pediatric EBV + PTCL cases. This table shows the clinical features and outcomes of the published pediatric EBV + PTCL cases. (DOCX 14 kb)

Additional file 5: **Table S2.** The clinicopathological difference in ENKTL, STLC and our case. This table shows 2 main kinds diseases which should be considered as differential diagnosis and summarizes the key points in differentiation. (DOCX 15 kb)

## Abbreviations

18F–FDG: 2-Deoxy-2-fluoro-D-glucose
ALCL: Anaplastic large cell lymphoma
CCD: Coiled-coiled domain
DBD: DNA binding domain
DS: Deauville 5-point score
EBV + PTCL: Epstein-Barr virus positive peripheral T cell lymphoma
FFPE: Formalin-fixed paraffin-embedded
GH-IGF-1: Growth hormone -insulin-like growth factor − 1
IGF-1: Insulin-like growth factor-1
L: Linker
LDH: Lactate dehydrogenase
ND: N-terminal domain
PCR-HA: Polymerase Chain Reaction heteroduplex analysis
PET-CT: Positron emission tomography–computed tomography
PTCL-NOS: Peripheral T cell lymphoma, not otherwise specific
SDS: Standard deviations
SH2: Src-homology 2
TAD: Transactivation domain
