# Supplementary figures and images for: Epstein-Barr virus positive peripheral T cell lymphoma with novel variants in STAT5B of a pediatric patient: a case report
Source: BMC Cancer. 2018 Apr 3;18:373. doi: 10.1186/s12885-018-4311-z (PMC5883291; doi:10.1186/s12885-018-4311-z)

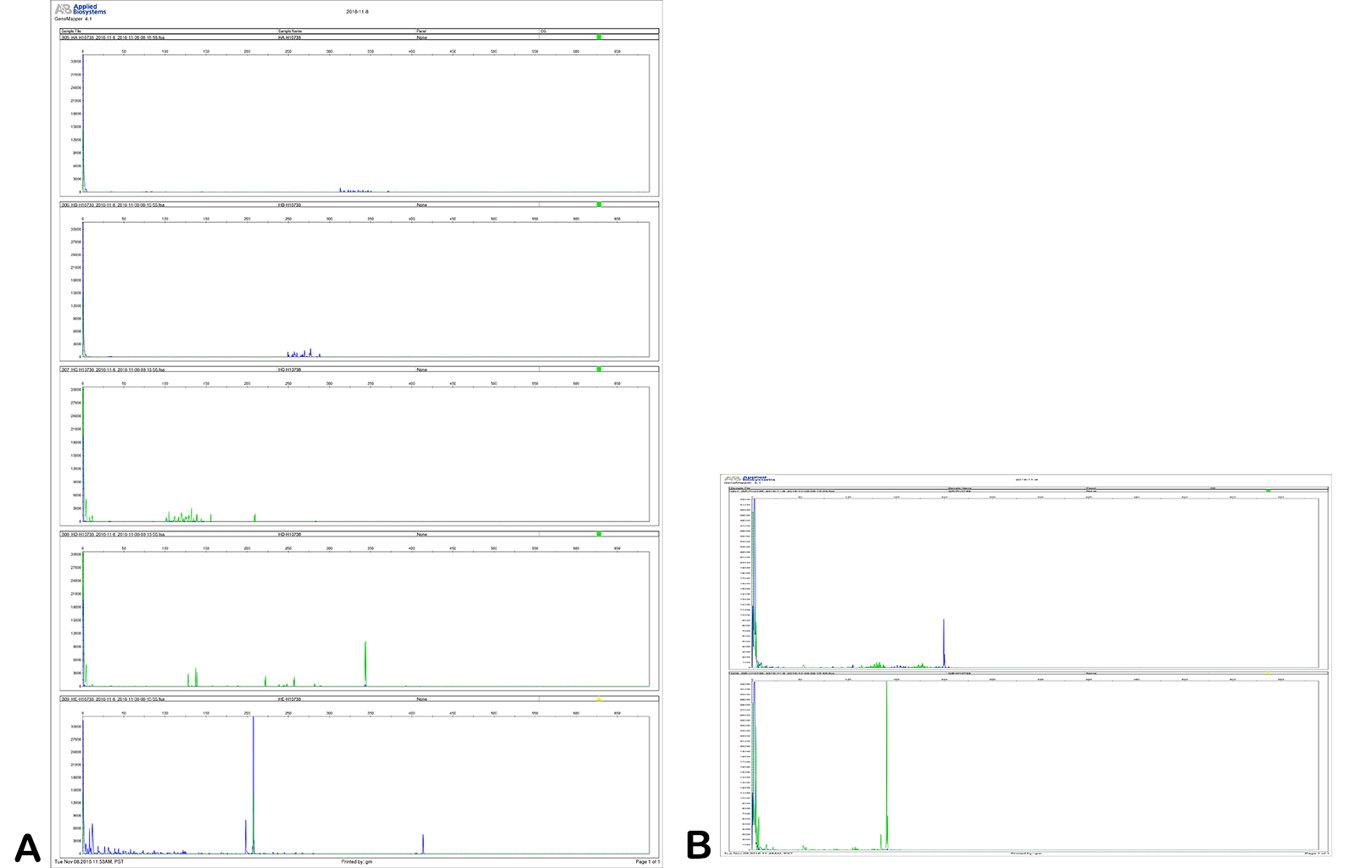

Supplement: Supplementary file 1 — Figure S1. IgH, IgK and TCR rearrangement. This figure shows results of IgH, IgK and TCR rearrangement. A: IgH and Igκ testing: No obvious clonal peak was detected (The peak in blue is normal according to the testing instruction). B: TCRγ testing: Clonal peak was detected (The peak in green). (TIFF 4875 kb) [file 12885_2018_4311_MOESM1_ESM.tif]

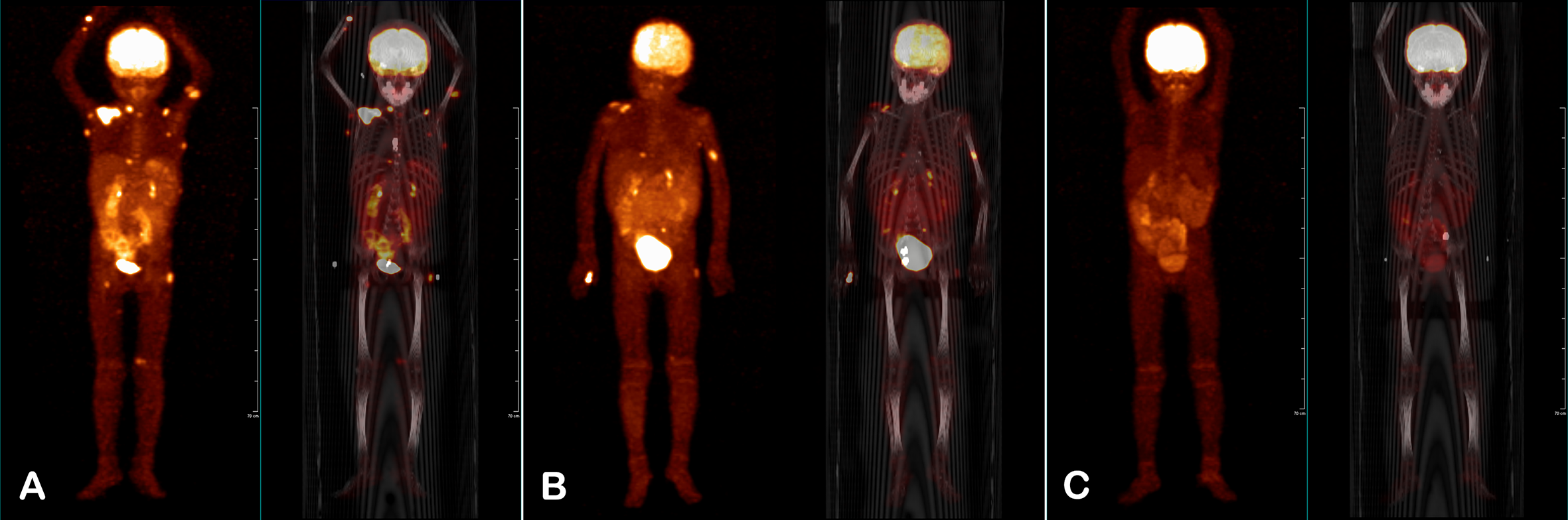

Supplement: Supplementary file 3 — Figure S2. PET-CT images of the patients. This figure shows PET-CT results of the patient (pre-treatment, after 2 cycles of chemotherapy and after 8 cycles of chemotherapy). A: Pre-treatment PET-CT showed multiple lesions in subcutaneous tissue, muscle et al. B: PET-CT after 2 cycles of SMILE regimen, DS = 5. C: PET-CT after 8 cycles of modified SMILE regimen, complete remission. (TIFF 5450 kb) [file 12885_2018_4311_MOESM3_ESM.tif]
