# Supplementary material for: Epstein-Barr virus positive peripheral T cell lymphoma with novel variants in STAT5B of a pediatric patient: a case report
Source: BMC Cancer. 2018 Apr 3;18:373. doi: 10.1186/s12885-018-4311-z (PMC5883291; doi:10.1186/s12885-018-4311-z)
Supplement: Supplementary file 2 — STAT5B variant investigation by Sanger Sequencing. This file provides the method of STAT5B variant investigation by Sanger Sequencing in details. (DOCX 13 kb) [file 12885_2018_4311_MOESM2_ESM.docx]

***STAT5B* variant investigation by Sanger Sequencing**

STAT5B variant analysis was performed on both formalin-fixed paraffin-embedded (FFPE) neoplastic tissue and blood sample. QIAamp DNA FFPE Tissue Kit (Qiagen Inc., Valencia, CA) and TIANamp Blood DNA Kit (DP318) were used to extract the genomic DNA. PCR primers were designed and synthesized referring to STAT5B sequence (RefSeq: NM_012448; CCDS:CCDS11423). The expected amplicons were recovered by AxyPrep DNA Gel Extraction Kit，AXYGEN (AP-GX-250). Gene sequencing was performed referring to instruction of BigDye® Terminator v3.1 Cycle Sequencing Kit and the results were analyzed by Applied Biosystems 3730xl DNA Analyzers (Applied Biosystems, Foster, CA).
