# Supplementary material for: Epstein-Barr virus positive peripheral T cell lymphoma with novel variants in STAT5B of a pediatric patient: a case report
Source: BMC Cancer. 2018 Apr 3;18:373. doi: 10.1186/s12885-018-4311-z (PMC5883291; doi:10.1186/s12885-018-4311-z)
Supplement: Supplementary file 4 — Table S1. Summary of pediatric EBV + PTCL cases. This table shows the clinical features and outcomes of the published pediatric EBV + PTCL cases. (DOCX 14 kb) [file 12885_2018_4311_MOESM4_ESM.docx]

Table s1: Summary of pediatric EBV+PTCL cases

| Reference | Age/Sex | Site | CM | CD8 | CD30 | CD56 | Diagnosis | Treatment | Outcome |
| --- | --- | --- | --- | --- | --- | --- | --- | --- | --- |
| This study | 9y/M | Multiple subcutaneous masses | + | + | + | - | EBV+PTCL | SMILE | Twice relapse in 6 months |
| [4] | 20mo/M | LN | + | + | - | - | EBV+PTCL | CHOEP | Clinically stable; 8 months after diagnosis |
| [5] | 5y/F | LN,BM,LV,SP | + | + | - | - | EBV+PTCL | Steroid | 20 days dead of disease |
| [6] | 7y/M | LN | NR | + | - | - | EBV+PTCL | None | Disease free for 80 months |

EBV+PTCL, EBV positive peripheral T-cell lymphoma; CM, cytotoxic molecule (including granzymeB and TIA-1); LN, lymph node; BM, bone marrow; LV, liver; SP, spleen; NR: not report
