# Supplementary material for: Epstein-Barr virus positive peripheral T cell lymphoma with novel variants in STAT5B of a pediatric patient: a case report
Source: BMC Cancer. 2018 Apr 3;18:373. doi: 10.1186/s12885-018-4311-z (PMC5883291; doi:10.1186/s12885-018-4311-z)
Supplement: Supplementary file 5 — Table S2. The clinicopathological difference in ENKTL, STLC and our case. This table shows 2 main kinds diseases which should be considered as differential diagnosis and summarizes the key points in differentiation. (DOCX 15 kb) [file 12885_2018_4311_MOESM5_ESM.docx]

Table s2: clinicophathological difference in ENKTL, STLC and our case

|  | **ENKTL** | **STLC** | **Our case** |
| --- | --- | --- | --- |
| **Onset age** | Adult  (median age 46y) | Children and young adults | 9 years old |
| **Site of involvement** | Extranodal involvement  (Upper aerodigestive tract, Skin, GI tract ) | Lymph node  Liver, Spleen, BM | Subcutaneous tissue, muscle and peripheral nerve system  No lymph node involvement |
| **Morphology** | Angiocentric and angiodestructive growth pattern with extensive necrosis  Medium-sized cells or mixture of small and large cells, cucumber-like cells  pleomorphic | Preserved architecture,  B-cell areas are depleted  Small or medium-sized cells  Lack substantial cytological atypia | Diffuse pattern  Centroblastoid |
| **Phenotype** |  |  |  |
| CD3 | + | + | + |
| CD4 | 4% positive | +(from CAEBV) | - |
| CD8 | 29% positive | +(from primary EBV infection) | + |
| CD30 | 50% positive | Unknown | + |
| CD56 | 67% positive | - | - |
| GB | + | + | + |
| TIA-1 | + | + | + |
| **EBER-ISH** | + | + | + |

ENKTL: extranodal NK/T cell lymphoma; STLC: systemic EBV+T-cell lymphoma of childhood; GI: gastrointestinal tract; CAEBV: chronic active EBV infection; BM: bone marrow, GB: granzyme B, TIA-1: T-cell intracellular antigen-1, EBER-ISH: In situ hybridization for EBV encoded RNA
